# SEC23A Inhibit Melanoma Metastatic through Secretory PF4 Cooperation with SPARC to Inhibit MAPK Signaling Pathway

**DOI:** 10.7150/ijbs.60866

**Published:** 2021-07-13

**Authors:** Bin Zeng, Zhiwei Sun, Qiting Zhao, Doudou Liu, Hao chen, Xiaoshuang Li, H. Rosie Xing, Jianyu Wang

**Affiliations:** 1 Institute of Life Sciences, Chongqing Medical University, Chongqing, China.; 2 State Key Laboratory of Ultrasound Engineering in Medicine Co-Founded by Chongqing and the Ministry of Science and Technology, School of Biomedical Engineering, Chongqing Medical University, Chongqing, China.

**Keywords:** melanoma, Sec23a, Pf4, Sparc, MAPK

## Abstract

Metastasis of melanoma to the distant organs is a multistep process in which the tumor microenvironment (TME) may play an important role. However, the relationship between metastatic progression and TME is intricate. In the present study, using melanoma derivative cell lines OL (oligometastatic) and POL (polymetastatic) that differ in their metastatic colonization capability, we have elucidated a new mechanism involving “SEC23A-PF4-MAPK/ERK axis” in which PF4 transported by COPII hinders metastasis through inhibition of MAPK/ERK signaling pathway. Furthermore, SPARC can act cooperatively to enhance the inhibition of Pf4 on ERK phosphorylation and melanoma cell metastasis. Our findings show the possibility of targeting cancer cell secretome for therapeutic development.

## Introduction

Cutaneous melanoma (CM) is a key form of malignancy of the skin^1^. According to the World Health Organization, approximately 132,000 cases of melanoma are diagnosed every year worldwide^2^. It is largely responsible for the skin cancer related death 1, 3. Conventional therapies have not been effective for the treatment of metastatic melanoma. Targeted therapy and immunotherapy show promises for advanced cutaneous melanomas*.* Nevertheless, acquired resistance to immunotherapy and targeted therapy remains a problem 4-6. Given these clinical challenges, it is urgent to identify new prognostic markers and to elucidate new anti-metastatic mechanisms that can be utilized for therapeutic development for melanoma.

The tumor microenvironment (TME) consists of not only tumor cells but also extracellular matrix, stroma, vascular networks, lymphatic networks and different types of immune cell^7^, ^8^. Tumor cells, by secreting a variety of cytokines, recruit stromal cells into the TME^9^. Although the interactions between cancer cells and TME in cancer progression have long been appreciated, the underlying mechanisms are complex and intricate. Accumulating evidence indicates that protein secreted by the tumor cell can influence tumor progression trait^10^-^12^.

SEC23A is involved in the assembly of the coat protein complex II (COPII) and mediates the translocation of most secretory proteins and lipids from the endoplasmic reticulum (ER) to the Golgi apparatus inside the cells^13^. Thus, SEC23A is a key regulator of the secretome. In our previous study, miR-200c augments melanoma metastasis by targeting Sec23a^14^. However, mechanisms underlying anti-metastatic action of Sec23a remain elusive.

In our previous study, using the metastatic melanoma cell model, we created stable melanoma derivative cell lines with (1) oligometastatic (OL) phenotype *in vivo* which shows limited number of lung metastasis; and with (2) polymetastatic (POL) phenotype *in vivo* that shows multi-organ wide spread metastasis 15. Thus, OL and POL cells while both are metastatic, they differ in their colonization capability, which is the rate-limiting step for the development of macroscopic distant metastases. In the present study, using OL and POL cell lines and their respective *in vivo* models, we have elucidated a new mechanism by which the secretomes of tumor cells hinder metastasis through inhibition of MAPK/ERK signaling pathway. Specifically, platelet factor-4(PF4) transported by SEC23A, may cooperate with another secreted protein acidic and rich in cysteine (SPARC) to inhibit melanoma metastasis via inhibition of MEPK/ERK activation.

## Materials and Methods

### Cell lines and cell culture

GFP-labeled mouse melanoma cells were kindly provided by Dr. Robert Hoffman (University of California San Diego). Oligometastatic cell line (OL) and polymetastatic cell line (POL) were created from the isolation of mouse melanoma cells from the lung metastases and underwent 3 rounds of serial *in vivo* passages as we previously reported^15^. Cells were cultured in MEM medium (Hyclone) with fetal bovine serum (FBS) (Gibco) and 1% penicillin-streptomycin (Hyclone), and maintained at 37°C with 5% CO2.

### Concentration and purification of serum-free media

Cells were cultured with serum-free media for 24 h. The conditioned media were collected and centrifuged at 3,000 × g for 5 min to remove dead cells and debris.An ultrafiltration tube (3kDa, Millipore) was used for further concentration, purification and then lyophilized.

### Lentivirus production and RNA interference

The shRNA targeting Sec23a sequences was 5′-GGAAGCTACAAGAATGGTTGT-3′. Sec23a overexpression (OE) plasmid (Plasmid #36158) and empty vector were obtained from Addgene. The shRNA targeting Pf4 sequences was 5′-ACACTTAACGGAGAGCCTG-3′. The lentivirus particles of shSec23a and Sec23a-OE were prepared by Sangon Biotech Co. The lentivirus particles of Pf4 were prepared by GenePharm co. A panel of 2 Sparc-siRNAs (siRNA-KD1, siRNA-KD2 and NC-siRNA) was designed and synthesized by GenePharm co.

RNA interference was achieved by transfection of siRNA with Lipofectamine 2000 reagents (Invitrogen) according to the manufacturer's instructions. The si-Sparc sequences was 5′-GCAGAGGUGACUGAGGUAUTT-3′ (kD1) and 5′-GGACUUCGAGAAGAACUAUTT-3′ (kD2).

### Reverse transcription and quantitative real-time polymerase chain reaction (RT-qPCR)

Total RNA was isolated using TRIZOL (Takara) according to the manufacturer's instructions. PCR reactions were set up in a 10 µl reaction volume and performed following the manufacturer's instructions. The forward primer sequence and reverse primer sequence of Sec23a were AGTGGCGGAAGTCAGGATAC and GGCATTGGAAA TCTGGAGTG, respectively. The forward primer sequence and reverse primer sequence of GAPDH were TTCACCACCATGGAGAAGGC and TGAAGTCGCAGGAGACAACC, respectively. The forward primer sequence and reverse primer sequence of Pf4 were CTGCTTCTGGGCCTGTTGT and CTCCCATTCTTCAGGGTGGC, respectively. The forward primer sequence and reverse primer sequence of Sparc were CCCCTGCCAGAACCATCATT and GCTCAGTGTGGGACAGGTAC, respectively.

### Western blotting analysis

Total protein was extracted with RIPA buffer (Beyotime), and concentration determined by the BCA method (Cwbiotech). 40-50μg of protein was used to run on 10%, 12% and 15% polyacrylamide (Beyotime) gels and transferred to a PVDF membrane (Millipore). The membrane was blocked using QuickBlock™ Blocking Buffer (Beyotime, P0252). Primary antibodies were incubated overnight at 4°C; Then, the second antibody was added and incubated at room temperature for 1 hour. The following primary antibodies were used: anti-SEC23A (CST), anti-Tubulin (CST), anti-PF4 (Sigma-Aldrich), anti-SPARC (Proteintech), anti-Gapdh (Proteintech), anti-ERK1/2 (Upstate), anti-phospho-ERK/2 (Proteintech), anti-phospho-AKT(Sungen Biotech), anti-AKT(CST).

### Cck8 proliferation assay

The cell counting kit (CCK)-8 assay was used to quantify cell proliferation. Cells were plated in 96-well plates at a density of 1.5 × 10^3^ cells per well, and cell growth was measured for successive 6 days. The absorbance of each well at 450nm was measured with an enzyme-linked immunosorbent assay reader.

### Transwell migration and invasion assay

Transwell migration and Matrigel invasion assay used 8μm pore size Transwell inserts. 300μl serum-free medium with 3-5 × 10^4^ tumor cells was seeded into the upper chamber, and 650μl medium containing 10% serum was added in the lower chamber. The cells that migrated and invaded the lower surface of the membrane were fixed with cold methanol and stained with crystal violet.

### Cell colony formation assay

Cells were seeded into 6-well plates at a density of 200-250 cells/well and incubated for 7 days. The culture medium was replaced every 3 days. The resulting colonies that consist of more than 50 cells were fixed with methanol and thereafter stained with 0.25% crystal violet, and counted.

### Secretory protein profile analysis

Conditioned culture media samples were collected and immediately snap frozen. Liquid chromatography-tandem mass spectrometry (LC/MS/MS) analyses were performed by Shanghai QE Biotech Co., Ltd. (Shanghai, China).

### Animal experiments

BALB/cA-nude mice were purchased from the Experimental Animal Centre of Chongqing Medical University. Animal experiments were performed in accordance with the institutional animal welfare guidelines of the Chongqing Medical University. NOD/SICD mouses, 5 weeks old, were injected with 5×10^5^ tumor cells via the tail vein. Heart, lung, liver, kidney and spleen were collected at the indicated time points on day 29-30 post tumor cell inoculation when animals were sacrificed.

### Ethical approval

Animal experiments were approved by Chongqing Medical University committee for animal experiments. All experiments were performed in accordance with relevant guidelines and regulations.

### Statistical analysis

Statistical analyses were performed by Student's independent t-test and One-way ANOVA using GraphPad Prism 8 software (Graphpad Software). Results were presented as mean ± standard deviation (SD). Differences were considered statistically significant when p<0.001 (***); p<0.01 (**) and p<0.05 (*). All the data were repeated three times.

## Results

### Sec23a expression negatively regulates the migration and invasion of melanoma cells in vitro

**Fig 1:**
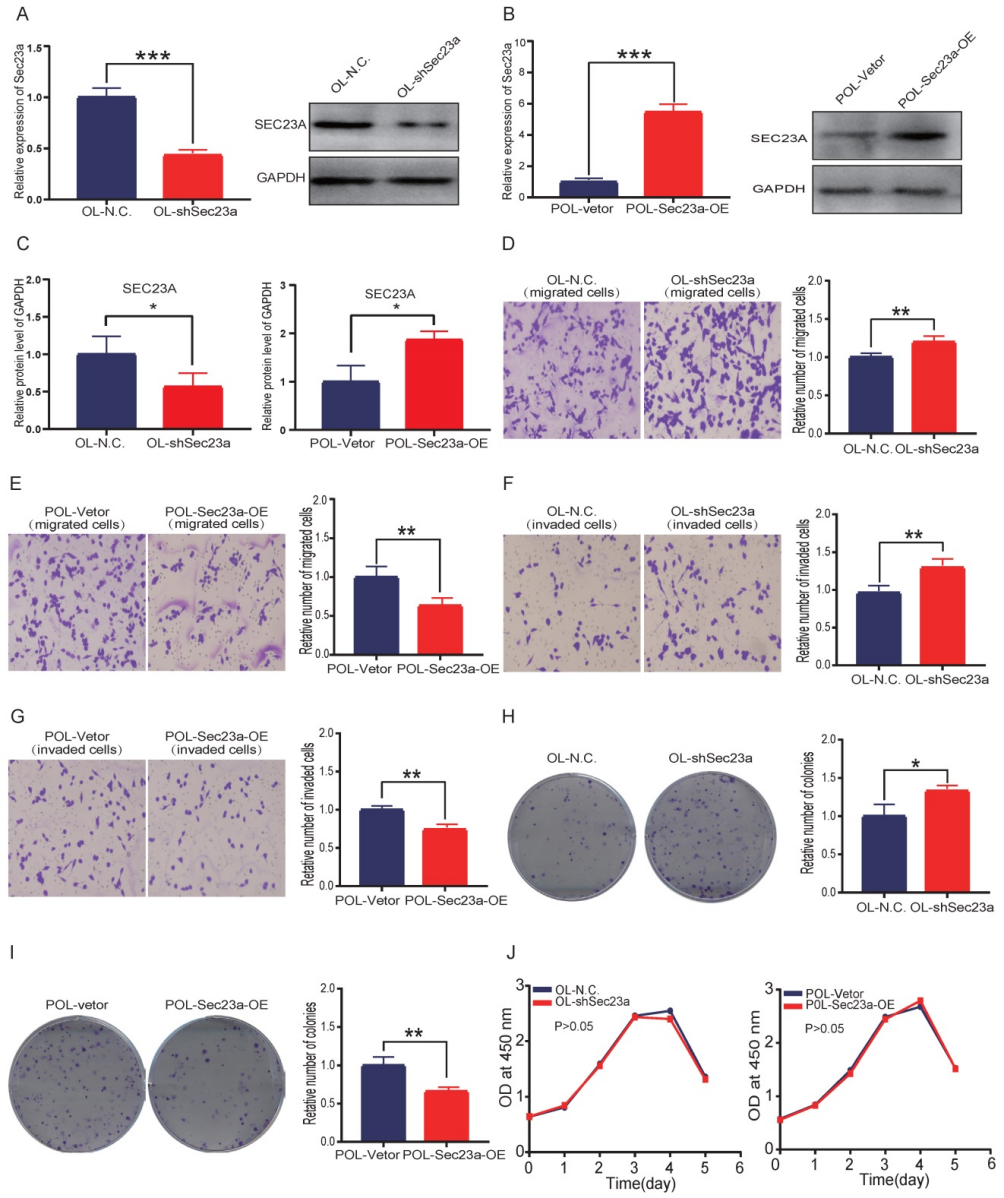
** Sec23a silencing significantly enhances the metastatic behavior of melanoma cells *in vitro*. (A, B)**, RT-qPCR and western blot were performed to confirm effective Sec23a silencing; **(C)** Quantitative analysis of the expressions of SEC23A in OL-N.C.,OL-shSec23a,POL-Vetor and POL-Sec23a-OE. **(D,F)** Sec23a knockdown significantly enhanced migration and invasion; **(E,G)** Overexpression of Sec23a inhibits invasion and migration of melanoma cells; **(H,I)** Effect of altered Sec23a expression on colony formation; **(J)** Cell proliferation was not affected by Sec23a expression changes, measured by CCK8 assay. (*p<.05,**p<.01,***p<.001).

Our previous study shows that OL and POL cell, while both are metastatic, but differ in their metastatic efficiency, in particular, the colonization efficiency. Further, the expression of Sec23a was higher in OL than POL cells^14^. Thus, our prior work suggests an inhibitory role of Sec23a in melanoma metastasis^14^. In this study, we first assessed the relationship between the Sec23a expression status and the metastatic behavior of melanoma cells *in vitro*. Sec23a was stably overexpressed in POL cells (POL-Sec23a-OE) in which endogenous SEC23A expression is lower than OL cells. In contrast, Sec23a was stably silenced in OL cell (OL-shSec23a) in which endogenous Sec23a expression is higher than POL cells. Overexpression or knockdown efficiency was confirmed by qPCR and WB, respectively (**Figure 1A-C**).

We next evaluated the effect of altered Sec23a expression on cell migration and invasion *in vitro* using the migration and Matrigel invasion assay, respectively (**Materials and Methods**). While silencing Sec23a in OL cells (OL-shSec23a vs OL-N.C.) augmented the invasiveness of OL cells (**Figure 1D and 1F**), overexpression of Sec23a in POL (POL-Sec23a-OE vs POL-Vetor) reduced invasiveness of POL cells (**Figure 1E and 1G**). We found the differences in cell migration and invasion were not due to alterations in cell proliferation by CCK8 assay (**Figure 1J**). Similarly, over-expression of Sec23a inhibited the clonogenicity of POL whereas Sec23a silencing enhanced the clonogenicity of OL cell, as quantified by the colony formation assay (**Figure 1H and 1I**). In summary, these observations show that Sec23a inhibits the metastatic ability of melanoma cells *in vitro*.

### Reduced Sec23a expression inhibits the secretion of PF4

**Figure 2:**
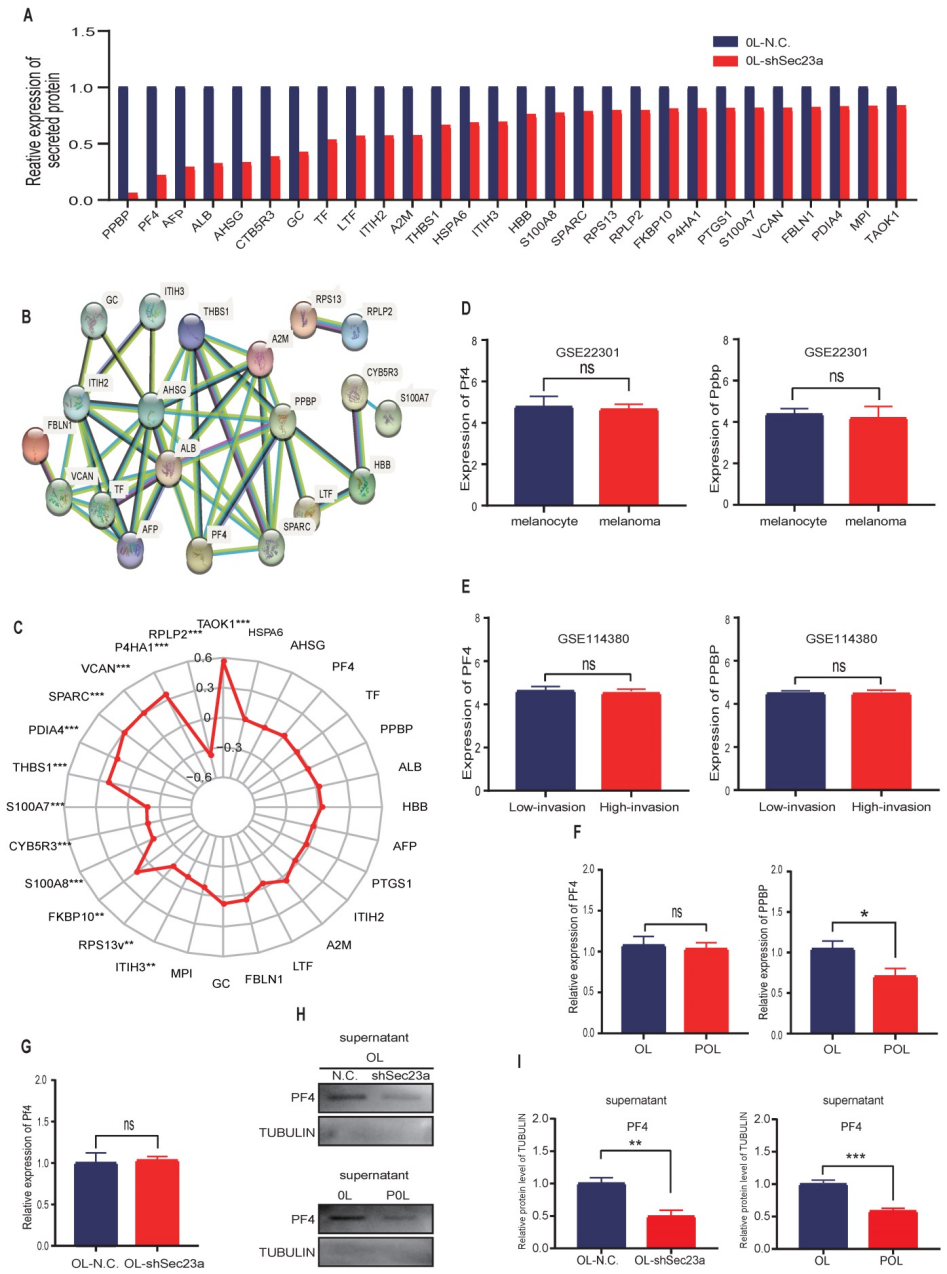
** Mass spectrometry (MS) detection of differentially secreted proteins in OL-N.C. and OL-shSec23a supernatant. (A)** quantification of differentially down-regulated secretory proteins; **(B)** STRING database analysis derived protein-protein interaction (PPI) network of the 28 down-regulated secretory proteins; **(C)** correlation of 28 down- regulated secretory proteins and Sec23a from TCGA analysis of melanoma datasets; **(D)** expression of Pf4 and Ppbp between melanoma cells and melanocyte in GSE22301; **(E)** expression of Pf4 and Ppbp between low- and high-metastatic melanoma cells in GSE114380**; (F)** expression of Pf4 and Ppbp between in OL vs POL cells; **(G)** expression of Pf4 in OL vs OL-shSec23a; **(H)** validation of the Pf4 down-regulation in OL-shSec23a and POL cells using conditioned medium, respectively. **(I)** Quantitative analysis of the expressions of Pf4 in the supernatants of OL-N.C.,OL-shSec23a,OL and POL cells (*p<.05,**p<.01,***p<.001).

Mass spectrometry (MS) detection of secreted proteins was performed using conditioned media of OL-N.C. cells and OL-shSec23a cells. A crude comparison resulted in 28 proteins that had reduced level of secretion in OL-shSec23a cells in which Sec23a was silenced (**Figure 2A**). PPI network analysis (https://string-db.org/) revealed protein-protein interaction of the 28 down-regulated secretory proteins (**Figure 2B**). Next, we evaluated the clinical relevance of the 28 down-regulated secretory proteins using the TCGA database. We excluded 13 secretory proteins that were regulated by Sec23a at transcriptional level (**Figure 2C**). Among the 15 proteins left, PF4 and pro-platelet basic protein (PPBP) secretion was most profoundly affected by Sec23a (**Figure 2A**). We compared Ppbp and Pf4 mRNA expression between melanoma and melanocyte (**Figure 2D**) and between the low-invasive and high-invasive melanoma (**Figure 2E**), respectively using GEO database. No significant differences were found (**Figure 2D and 2E**). We next measured Ppbp and Pf4 mRNA expression in POL and OL cells and found that while Ppbp was significantly decreased in POL cells where Sec23a expression was reduced^14^ compared to OL cells, transcription of Pf4 was largely unchanged in POL and OL cells (**Figure 2F**). Further, Pf4 mRNA expression remained unchanged in OL-shSec23a cells in which Sec23a was silenced (**Figure 2G**). These results indicate that decreased Ppbp level in the secretome of OL-shSec23a cells (**Figure 2A**) is most likely due to altered transcriptional expression. To confirm PF4 secretion is regulated by Sec23a, PF4 protein expression in the supernatants of POL and OL cells was determined by WB analysis. PF4 was significantly more abundant in the supernatant of OL cells than that of the POL cell (**Figure 2H and 2I***)*, corresponding to the expression of Sec23a in the two cell lines 14. These results demonstrate that PF4 secretion, not transcription is regulated by Sec23a.

### Pf4 knockdown promotes melanoma cell metastasis in vitro

**Figure 3:**
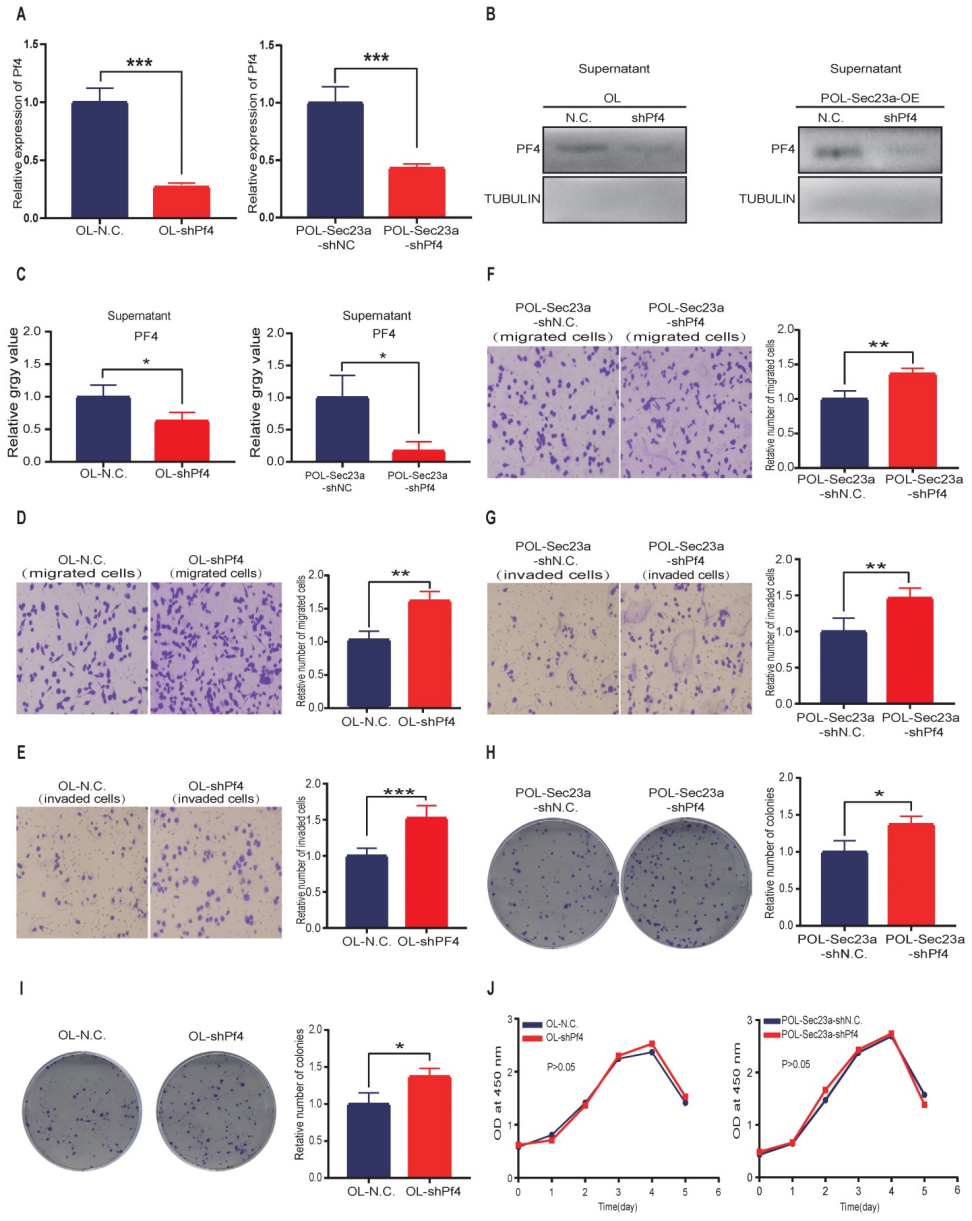
** Pf4 silencing in OL and POL-Sec23a-OE cells correlates with the invasive behavior of melanoma cells. (A)** Real-time PCR validation of Pf4 silencing; **(B)** western blot analysis of PF4 secretion in conditioned media upon Pf4 silencing; **(C)** Quantitative analysis of the expressions of PF4 in the supernatants of OL-N.C. ,OL-shPf4,POL-Sec23a-shNC and POL-Sec23a-shPf4. **(D-G)** Pf4 silencing significantly enhanced melanoma cell migration and invasion (**Material and Methods**); **(H, I)** Pf4 silencing significantly enhanced colony formation of melanoma cells; **(J)** Pf4 silencing did not alter cell proliferation, measured by CCK8 assay (*p<.05,**p<.01,***p<.001).

We next constructed stable Pf4 knock-down OL and POL-Sec23a-OE cell lines (OL-shPf4 and POL-Sec23a-shPf4), respectively (**Figure 3A-3C**). Pf4 Knockdown enhanced the migration and invasion (**Figures 3D-3G**) and colony formation (**Figure 3H-3I**) in OL and POL-Sec23a-OE cells which was not due to alterations in cell proliferation rate (**Figure 3J**). In summary, our data collectively demonstrate that Pf4 inhibits metastatic capability of melanoma cells in vitro.

### Sec23a regulates melanoma metastasis by secreted PF4 in vitro and in vivo

**Figure 4:**
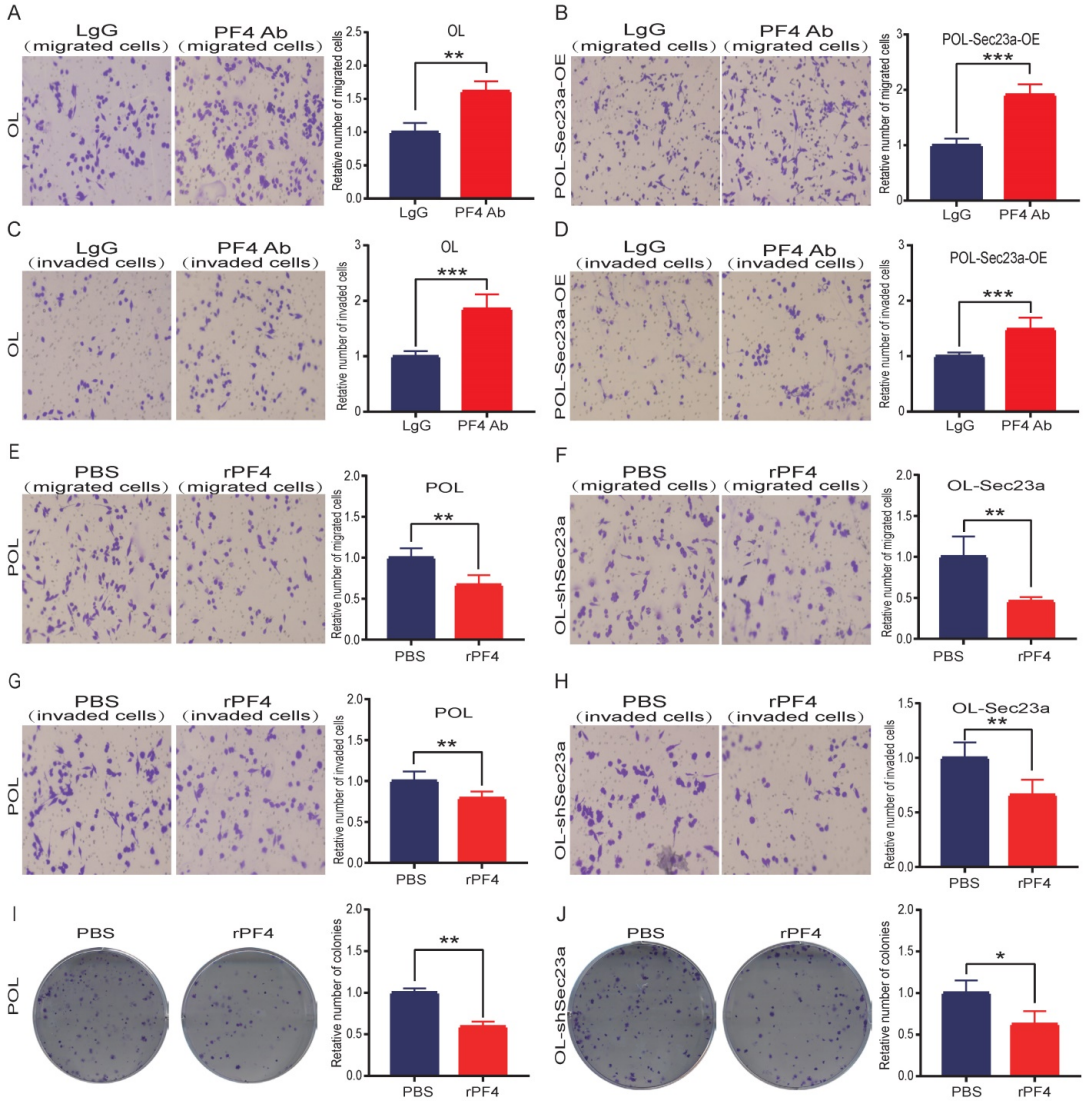
** Alteration of secreted PF4 affects the invasive behavior of melanoma cells.** Neutralization of PF4 with PF4 antibody (10ug/ml) reversed the inhibitory effect of Pf4 on OL (**A, C**) and POL-Sec23a-OE (**B, D**) cell migration and invasion; Recombinant PF4 protein (450 ng/ml) treatment inhibited POL cell (**E, G**); and augmented OL-shSec23a cell **(F, H)** migration and invasion**. (I, J)** Recombinant PF4 protein (450 ng/ml) treatment prevented Pf4 down regulation-induced increase in colony formation in POL and OL-shSec23a cells (*p<.05, **p<.01,***p<.001).

We next investigated whether the inhibitory effect of SEC23A on melanoma cell invasion is mediated by secreted PF4. PF4 secreted by OL cells and POL-Sec23a-OE cells was neutralized by treatment with 10ug/ml PF4 antibody for 24h; 10ug/ml rabbit lgG (Solarbio) was used as a negative control antibody. As expected, the inhibitory effects of Sec23a on OL cells and POL-Sec23a- OE cells migration and invasion were attenuated by PF4 neutralizing antibody treatment (**Figure 4A-4D**). In contrast, OL- shSec23a cells and POL cells, in which PF4 secretion was low, were treated with recombinant PF4 protein (rPF4) (450 ng/ml) for 24h.As a control, PBS was added instead of recombinant proteins. rPF4 treatment reversed the increase of migration and invasion of OL-shSec23a cell and POL cell due to Sec23a silencing or low expression, respectively (**Figure 4E-4H**). Similar results were seen in colony formation (**Figure 4I-4J**). Taken together, these results indicate that secreted PF4 acts downstream Sec23a to mediate its inhibition of melanoma cell migration and invasion *in vitro*.

**Figure 5:**
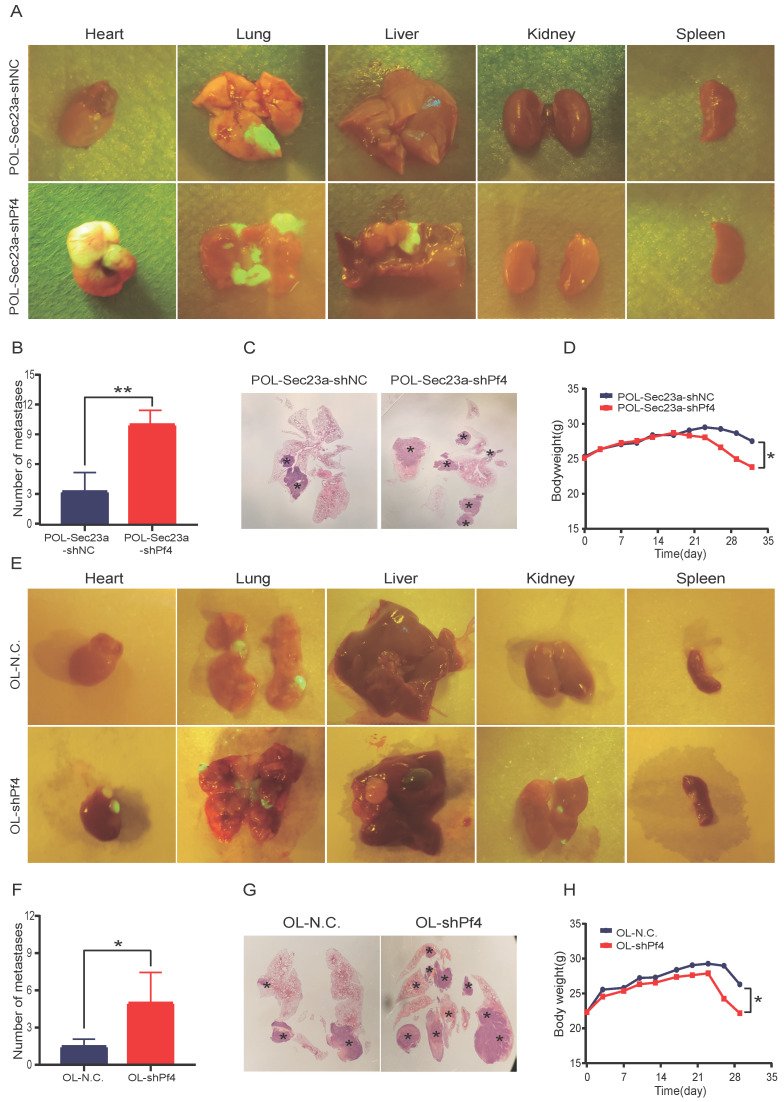
** Stable Pf4 silencing significantly enhances the metastatic capability of melanoma cells in NOD/SCID mice. (A, E)** Photographic representation of macroscopic metastases of NOD/SCID mice 4 weeks after tail vein injection of control and Pf4 knockdown cells (POL-Sec23a-OE-shPf4 and OL-shPf4) (Four mice per group); **(B, F)** Quantitative analysis of the surface metastatic foci in control and Pf4 knockdown cells; **(C,G)** representative whole-lung images to visualize metastases on the lung (asterisk) by H&E staining; **(D,H)** body weight changes of mice receiving control and Pf4 knockdown cells (*p<.05,**p<.01,***p<.001).

To evaluate the role of Pf4 on melanoma metastatic progression *in vivo*, we inoculated OL-shPf4, POL-Sec23a-OE-shPf4 cells in which Pf4 was silenced and control cells to NOD/SCID mice by tail-vein injection. Four weeks later, the mice received OL-shPf4, POL-Sec23a-OE-shPf4 cells suffered from multiple organ metastasis (**Figure 5A and 5E**) (**Figure S2, A-B**). The number of the macroscopic metastatic foci on each involved organ were counted. Stable Pf4 silencing significantly promoted melanoma cell metastasis in NOD/SCID mice shifting from single-organ limited metastasis seen in the control group (OL-NC and POL-Sec23a-NC**)** to multi-organ extensive metastasis (**Figures 5A, 5C, 5E, 5G**). Meanwhile, metastatic foci in lungs in the Pf4 knock-down groups was significantly more than that in the control group (**Figure 5B and Figure 5F**). Mice inoculated with Pf4 knockdown cells suffered significantly more weight loss than that of the control cells (**Figure 5D and Figure 5H**). These results collectively demonstrate that Pf4 downregulation effectively promotes the metastatic progression of melanoma cells *in vivo*. Thus, Pf4 functions to inhibit the extend of melanoma metastasis.

### The inhibitory effect of Pf4 on melanoma cell migration and invasionin vitro is achieved via inactivation of the MAPK/ERK signaling

**Figure 6:**
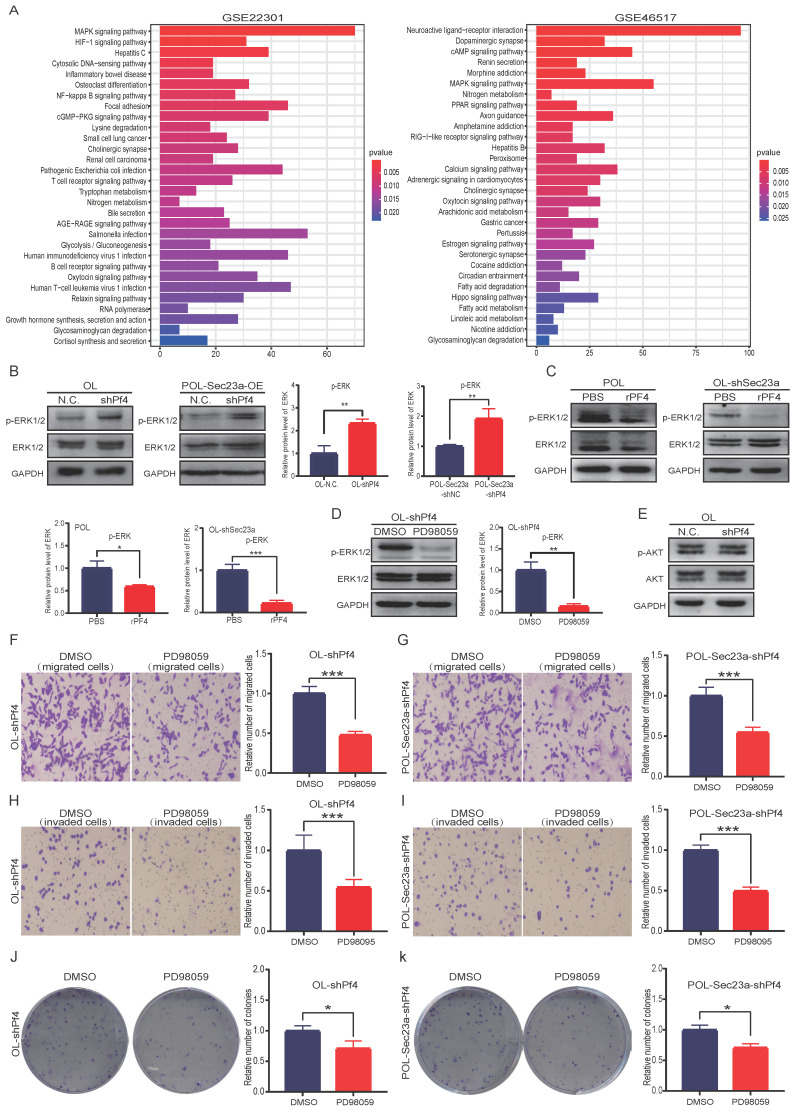
** Pf4 acts downstream of Sec23a to inhibit MAPK/ERK activation. (A)** KEGG pathway enrichment analysis for Pf4-related genes in GSE22301 and GSE46517 and prioritized MAPK/ERK pathway; **(B) (left)**Pf4 knockdown augmented ERK/MAPK activation in OL and POL-Sec23a-OE cells;** (right)** Quantitative analysis of the expressions of p-ERK in OL-N.C. ,OL-shPf4,POL-Sec23a-shNC and POL-Sec23a-shPf4 **(C) (top)** recombinant PF4 protein (450 ng/ml) treatment inhibited ERK/MAPK activation in OL-shSec23a and POL cell; **(bottom)** Quantitative analysis of the expressions of p-ERK in PBS and rPF4. **(D) (left)** PD98059 (20uM) treatment prevented the increase of p-ERK caused by Pf4 knockdown in OL-shPf4; **(right)** Quantitative analysis of the expressions of p-ERK in DMSO and PD98059. **(E)** Pf4 knockdown had no significant effect on AKT activation in OL cells; **(F-I)** PD98059 (20uM) treatment prevented the increase of OL cell invasion and migration caused by Pf4 knockdown (OL-shPf4 cells); **(J,K)** PD98059(20uM) treatment prevented the increase of colony formation in POL-Sec23a and OL cells caused by Pf4 knockdown (OL-shPf4 and POL-Sec23a-OE-shPf4 cells).(*p<.05, **p<.01,***p<.001).

The Pf4-related genes were obtained using the public microarray database GEO of human melanoma samples (https://www.ncbi.nlm.nih. gov/geo/). KEGG analysis was performed using the Pf4-related genes by the R software (**Figure 6A**). We focused our biological validation on the MAPK signaling pathway, which had the highest P value. Although it is one of the most studied intracellular signaling pathway in cancer 16, its molecular relationship with Pf4 has not been established. We conducted WB analysis to evaluate MAPK activation status** (Materials and Methods)** when Pf4 expression was altered. Pf4 silencing in OL- or POL-Sec23a-OE cells that have higher level of secreted Pf4, resulted in the significant enhancement of p-ERK while the expression of total ERK1/2 was not affected (**Figure 6B**). In contrast, treatment of Pf4 low POL- or OL-shSec23a cells with 450 ng/ml recombinant PF4 (rPF4) inhibited the activation of MAPK signaling pathway (**Figure 6C**). These observations indicate that Pf4 is upstream of MAPK pathway and negatively regulates its activation.

To confirm our findings that MAPK signaling is downstream of Pf4, we treated OL-siPf4 cells with ERK1/2 inhibitor PD98059 (20uM) and assayed ERK phosphorylation. PD98059 treatment effectively reversed the activation of ERK seen in OL-siPf4 cells (**Figure 6B and Figure 6D**). To determine the specificity of the regulatory effect of Pf4 on the MAPK pathway, we analyzed the activation status of PI3K-AKT signaling pathway, another well-established oncogenic intracellular signaling pathway. Pf4 silencing had no obvious effect on Akt phosphorylation in OL-siPf4 cells (**Figure 6E**). We next show that the inhibitory effect of secretory Pf4 on cell invasive properties and colony formation requires MAPK signaling in OL-siPf4 and POL-Sec23a-OE-siPf4 cells (**Materials and Methods, Figures 6F-6K).** Further, the changes in cell invasive behavior were not due to the effect of MAPK/ERK signaling on cell proliferation (**Figure S3A**).

In summary, our data show that secretory Pf4 inhibits the metastatic progression of melanoma cells via inactivation of the MAPK signaling pathway.

### Sparc cooperate with Pf4 to inhibit the metastatic behavior of melanoma cells in vitro

**Figure 7:**
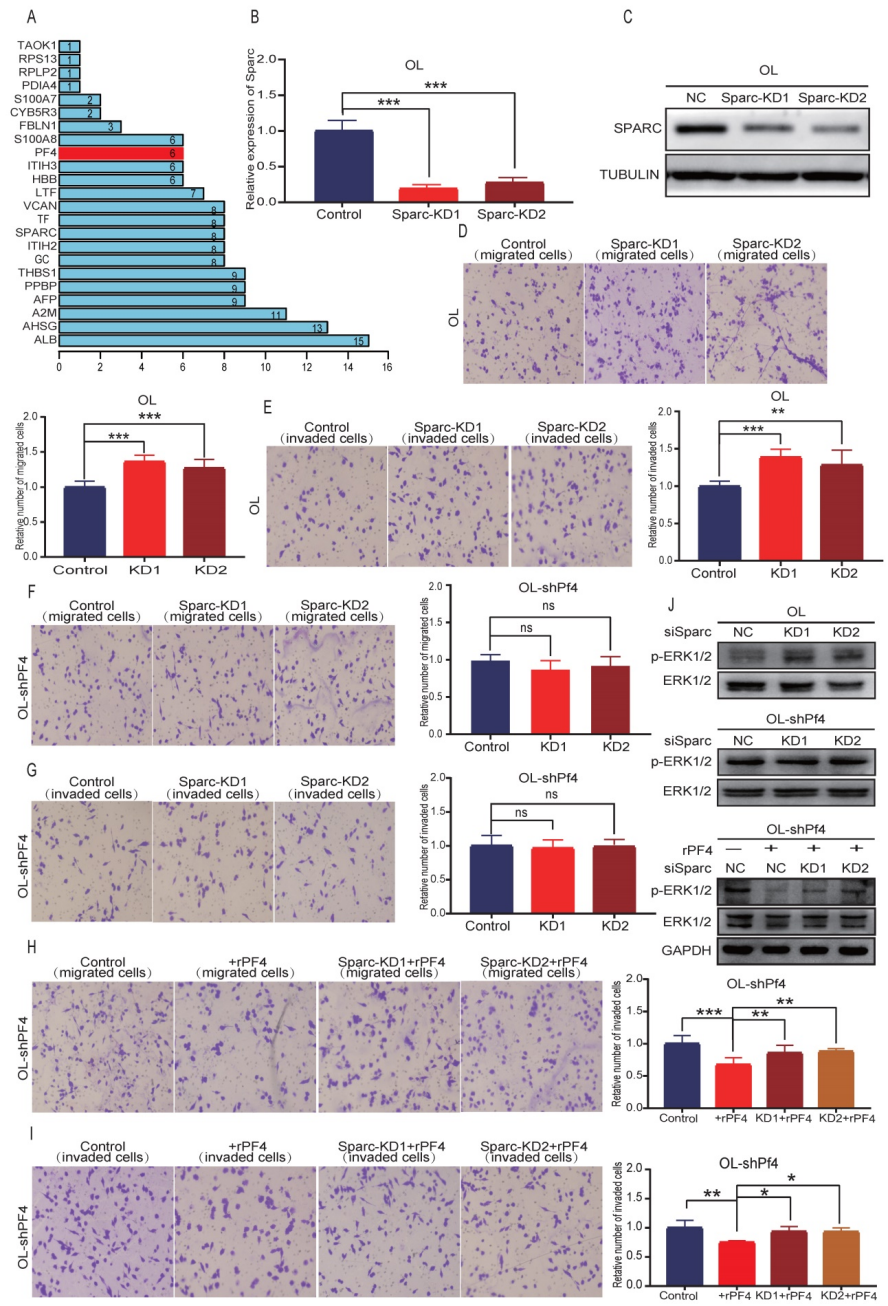
** Sparc cooperates with Pf4 to augment the inhibition of Pf4 on the invasive behavior of melanoma cells *in vitro*. (A)** the bar plot of PPI protein-protein interaction of 28 differentially down-regulated secretory proteins. SPARC, PPBP, ALB, AHSG, A2M and THBS1 were predicted to bind to PF4; melanoma cells were transfected with scramble siRNA (control) or siRNA against Sparc. Effective Sparc silencing was confirmed by quantitative real-time PCR **(B)** and **(C)** WB analysis; **(D, E)** Sparc knockdown significantly enhanced OL cell migration and invasion *in vitro*; **(F, G)** Sparc knockdown did not change the invasion and migration of OL cells in the absence of Pf4 (OL-shPf4); **(H, I)** Sparc knockdown attenuated the inhibitory effect of recombinant PF4 protein (450 ng/ml) on OL-shPf4 cells migration and invasion; **(J)** (**top**) Sparc knockdown enhanced p-ERK in OL cells; (**center**) Sparc knockdown had no effect on p-ERK in OL-shPf4 cells in which Pf4 was stably silenced; (**bottom**) Sparc attenuated the inhibition of rPf4 protein (450 ng/ml) on the levels of p-ERK in OL-rhPf4 cells (*p<.05, **p<.01,***p<.001).

In a separate line of thinking, we hypothesize that SEC23A-regulated secretory proteins may act cooperatively to change the oncogenic properties of the cancer cells. To test this possibility, we performed literature analysis of the 28 down-regulated secretory proteins for validated interactions. Literature analysis revealed that among the 28 down-regulated secretory proteins, Pf4 and thrombospondin 1 (THBS1) can bind to heparin^17^, ^18^. SPARC, which is among the 28 downregulated secretory proteins, is an acidic protein like heparin^19^. Thus, Pf4 and THBS1 may bind to SPARC. PPI analysis predicted the binding of Pf4 with SPARC (**Figure 7A**). Additional proteins that were predicted to bind to PF4 are PPBP, ALB, AHSG, A2M and THBS1 (**Figure 7A**).

To test the hypothesis that PF4 and SPARC may act cooperatively to inhibit melanoma metastasis, Sparc expression was silenced using siRNA (**Figure 7B**). To rule out nonspecific interference, we constructed 2 different sequences of siRNA oligonucleotides for Sparc (**Figure 7B-7C**) (**Figure S1A**). Effective Sparc silencing did not affect OL cell proliferation (**Figure S3B**), but augmented migration (**Figure 7D**), invasion (**Figure 7E**) and MAPK/ERK activation of OL cells (**Figure 7J, upper panel**) (**Figure S1, B-left**). In OL-shPf4 cells in which Pf4 was silenced, we found that Sparc silencing had no significant effect on migration (**Figure 7F**), invasion (**Figure 7G**) and pERK (**Figure 7J**, **center panel**; **Figure S1,B-right**). However, when secreted Pf4 was added back through treatment with recombinant Pf4 (rPF4), Sparc knockdown partially reversed the inhibitory effect of recombinant PF4 (rPF4) on OL-shPf4 cell migration, invasion and MAPK/ERK activation (pERK level) (**Figure 7H, 7I and 7J-lower panel**). These observations show that proteins in the secretome, in this case, SPARK and PF4 may act cooperatively to achieve even more pronounced attenuation of melanoma metastatic progression. The mechanisms underlying such cooperative activity merits future investigation.

## Discussion

Metastasis occurs through a complex multi-step process, and is subjected to the interplay of a plethora of factors^20^. Tumor microenvironment (TME) plays a crucial role in cancer treatment responses and metastasis^21^, ^22^. One hypothesis is that secreted proteins produced by the cancer cells affects metastatic colonization, the rate limiting step of metastasis, by remodeling TME^23^. In the present study, using OL and POL cell lines and their respective *in vivo* models, we have elucidated a new mechanism by which the secretory proteins in the secretome of tumor cells hinders metastasis through inhibition of MAPK/ERK signaling pathway.

Previous studies of secretory proteins focused primarily on the changes occurred at the transcriptional level. Given that SEC23A is involved in COPII assembly, down regulation of the 28 secretory proteins in OL cells upon Sec23a silencing occurred due to inhibition of Sec23a regulated secretory pathway.

The most important finding of the current study is that PF4 transported by SEC23A, may cooperate with another secretory protein SPARC to inhibit melanoma metastasis via inhibition of MEPK/ERK activation. We made the following three observations that had not been previously reported:

**First, the inhibitory effect of Sec23a on melanoma metastasis is mediated by secreted PF4**. We prioritized Pf4 for in depth characterization based on the following: (1) Pf4 was one of the most down-regulated secreted proteins upon Sec23a silencing; (2) Sec23a had no effect on Pf4 transcription; (3) there was no significant difference in Pf4 mRNA expression in OL and POL cells that differ in invasive behavior. These results indicate that changes of Pf4 protein in the supernatant were largely dependent on Sec23a‐regulated transport pathways.

PF4 is mainly produced by platelets and is involved in blood coagulating, wound repair and inflammatory^24^. In our study, functional *in vitro* and *in vivo* experiments have shown that non-platelet-derived secretory PF4, produced by melanoma cancer cells, inhibits metastatic progression. Our data was in agreement with the study of Fang^25^ and Struyf^26^. However, pro-metastasis function of PF4 has also been reported in lung^27^ and colon cancer^28^, ^29^. Upon close examination of the literature, it appears that non-platelet derived PF4, when acts on stromal cells (such as immune cells and endothelial cells) in TME, will function as a promoter of metastatic progression. On the other hand, when it acts on cancer cells, it is anti-metastatic, as we reported here.

**Second, MAPK/ERK signaling mediates the inhibitory effect of secreted PF4 on the invasive behavior of melanoma cells**. We conducted bioinformatics analysis of GEO database (GSE22301 and GSE46517) to derive Pf4-related genes. KEGG pathway analysis was performed on Pf4-related genes and identified the MAPK signaling pathway as a potential downstream effector of Pf4 in melanoma. Previous literature showed that the inhibitory effect of Pf4 on endothelial cell proliferation 30 was achieved through inhibition of ERK phosphorylation. We conducted experiments and show that MAPK/ERK signaling mediates the inhibitory effect of secreted PF4 on the invasive behavior of melanoma cells.

**Third, secretory SPARK and PF4 in the secretome may act cooperatively to achieve even more pronounced attenuation of melanoma metastatic progression.** In a separate line of thinking, we hypothesized that Sec23a-regulated secretory proteins may act cooperatively to change the oncogenic properties of the cancer cells. Here we show that PF4 and SPARC in the Sec23a-regulated secretome can act cooperatively to inhibit melanoma metastasis. In the biological validation experiments we conducted, in the absence of Pf4, Sparc had no effect on the invasive behavior of melanoma cells *in vitro*. However, when Pf4 status altered in melanoma cells, Sparc acted cooperatively to enhance the effect of Pf4. A previous study in the literature reported the oncogenic effect of Sparc on melanoma cell proliferation and survival 31. In our melanoma cell derived metastatic OL and POL cells that differ in metastatic colonization capability, Sparc appears to inhibit metastatic colonization in the presence of Pf4. To our knowledge, this is the first evidence that proteins in the cancer cell produced secretome can act cooperatively to modify cancer cell traits. The mechanisms underlying such cooperative activity merits future investigation.

In conclusion, this study is the first to illustrate that secretome produced by the cancer cells can affect metastatic colonization, the rate limiting step of metastasis via changing the quantity of secretory proteins into the TME. Further, the secretory proteins in the cancer cell-regulated secretome can work cooperatively to inactivate MAPK/ERK leading to more pronounced inhibition of melanoma metastasis. We have identified a new mechanism underlying the inhibitory effect of Sec23a on cancer metastasis. Our work presented here has provided a new theoretical framework for developing new anti-metastatic strategies that aim at TME. Whether non-platelet derived secreted PF4 can be used as a favorable prognostic marker merits clinical investigation and validation.

## Supplementary Material

Supplementary figures.
