# Supplementary material for: SEC23A Inhibit Melanoma Metastatic through Secretory PF4 Cooperation with SPARC to Inhibit MAPK Signaling Pathway
Source: Int J Biol Sci. 2021 Jul 13;17(12):3000–12. doi: 10.7150/ijbs.60866 (PMC8375231; doi:10.7150/ijbs.60866)
Supplement: Supplementary file 1 — Supplementary figures. [file ijbsv17p3000s1.pdf]

Supplementary Fig. S1

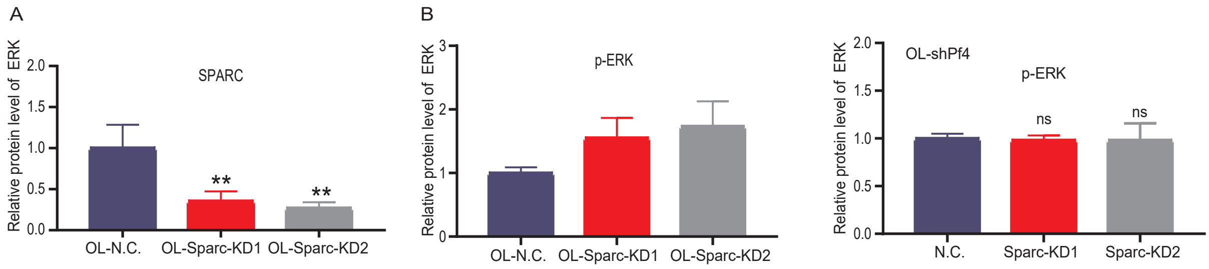

Supplementary Figure S1. (A) Quantitative analysis of the expressions of SPARC in OL-N.C. and OL-siSparc-KD cells. (B) Quantitative analysis of the expressions of p-ERK in OL-N.C. , OL-siSparc-KD, OL-shPf4 and OL-shPf4-siSparc-KD cells (*p<.05, **p<.01,***p<.001).

Supplementary Fig. S2

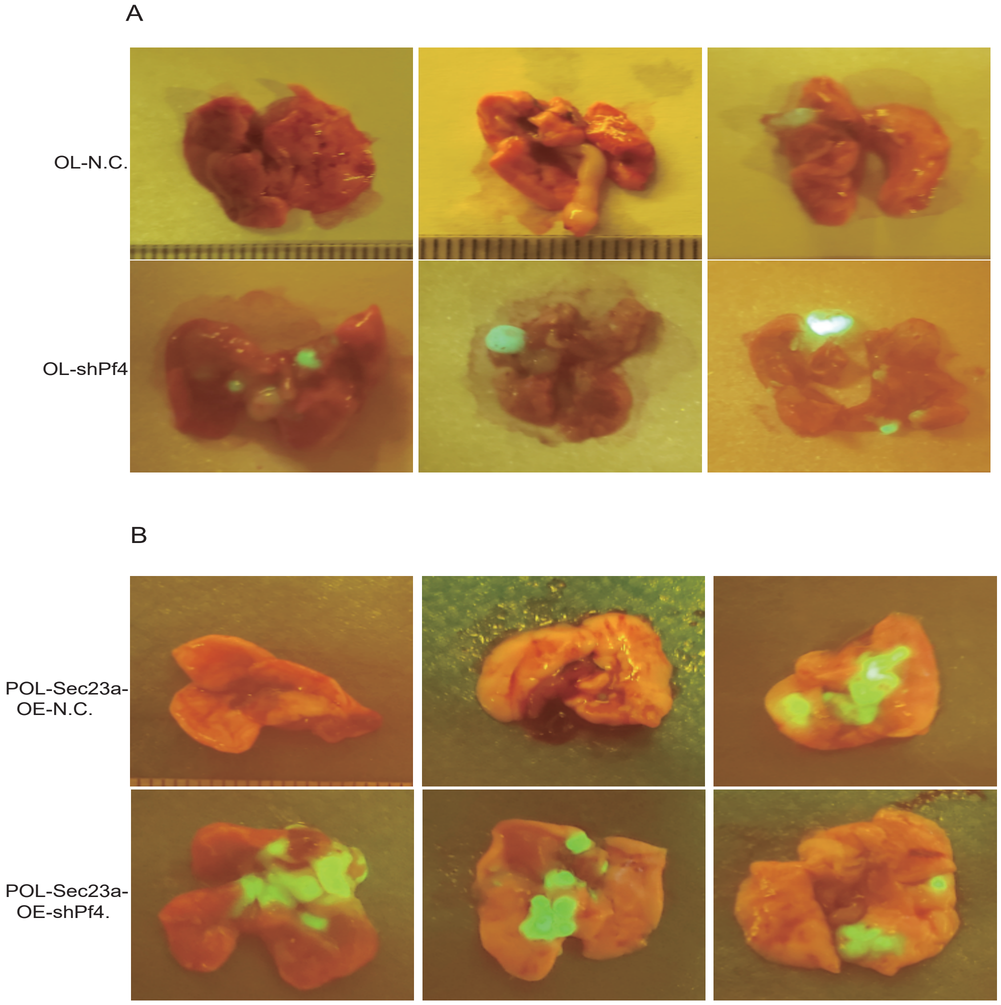

Supplementary Fig. S2 (A, B) Photographic representation of lung metastases of NOD/SCID mice 4 weeks after tail vein injection of control and Pf4 knockdown cells.

Supplementary Fig. S3

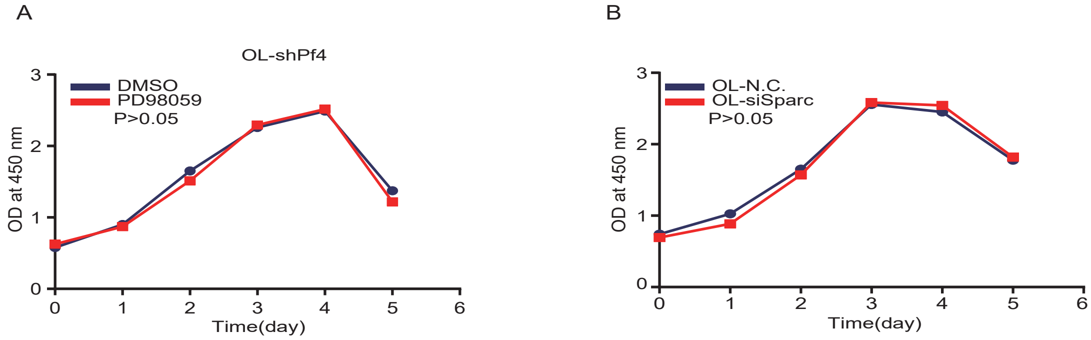

Supplementary Figure S3. Cell proliferation was measured by CCK8 assay.
(A) PD98059 treatment had no significant effect on OL-shPf4 cell proliferation.
(B) Sparc silencing had no significant effect on OL cell proliferation.(*p<.05, **p<.01,***p<.001).
